# A New Analysis of Archaea–Bacteria Domain Separation: Variable Phylogenetic Distance and the Tempo of Early Evolution

**DOI:** 10.1093/molbev/msaa089

**Published:** 2020-04-21

**Authors:** Sarah J Berkemer, Shawn E McGlynn

**Affiliations:** m1 Max Planck Institute for Mathematics in the Sciences, Leipzig, Germany; m2 Bioinformatics Group, Department of Computer Science, University Leipzig, Leipzig, Germany; m3 Competence Center for Scalable Data Services and Solutions, Dresden/Leipzig, Germany; m4 Earth-Life Science Institute, Tokyo Institute of Technology, Meguro, Tokyo, Japan; m5 Blue Marble Space Institute of Science, Seattle, WA; m6 RIKEN Center for Sustainable Resource Science (CSRS), Saitama, Japan

**Keywords:** LUCA, conserved orthologous groups of proteins, orthology, microbial physiology, progenote

## Abstract

Comparative genomics and molecular phylogenetics are foundational for understanding biological evolution. Although many studies have been made with the aim of understanding the genomic contents of early life, uncertainty remains. A study by Weiss et al. (Weiss MC, Sousa FL, Mrnjavac N, Neukirchen S, Roettger M, Nelson-Sathi S, Martin WF. 2016. The physiology and habitat of the last universal common ancestor. *Nat Microbiol.* 1(9):16116.) identified a number of protein families in the last universal common ancestor of archaea and bacteria (LUCA) which were not found in previous works. Here, we report new research that suggests the clustering approaches used in this previous study undersampled protein families, resulting in incomplete phylogenetic trees which do not reflect protein family evolution. Phylogenetic analysis of protein families which include more sequence homologs rejects a simple LUCA hypothesis based on phylogenetic separation of the bacterial and archaeal domains for a majority of the previously identified LUCA proteins (∼82%). To supplement limitations of phylogenetic inference derived from incompletely populated orthologous groups and to test the hypothesis of a period of rapid evolution preceding the separation of the domains, we compared phylogenetic distances both within and between domains, for thousands of orthologous groups. We find a substantial diversity of interdomain versus intradomain branch lengths, even among protein families which exhibit a single domain separating branch and are thought to be associated with the LUCA. Additionally, phylogenetic trees with long interdomain branches relative to intradomain branches are enriched in information categories of protein families in comparison to those associated with metabolic functions. These results provide a new view of protein family evolution and temper claims about the phenotype and habitat of the LUCA.

## Introduction

A longstanding goal of evolutionary biology is to infer the traits of the most ancient organisms. Conserved presence of a gene in a large number of archaea and bacteria can provide evidence of presence prior to the formation of these two domains, and if phylogenetic analysis indicates domain separation, presence in the last universal common ancestor of archaea and bacteria (LUCA) is predicted with greater confidence (Woese 1987; Woese et al. 1990; Harris et al. 2003; Koonin 2003; Charlebois and Doolittle 2004). Although molecular markers such as the 16s ribosomal RNA gene (Woese et al. 1990), ribosomal proteins (Hug et al. 2016), and some nucleotide polymerase subunits such as RpoB (Case et al. 2007) have indicated overall taxonomic relationships upon phylogenetic analysis, comparison of these molecules does not give insight into the metabolisms which power their host cells. To access traits other than those corresponding to these marker genes, gene or protein trees corresponding to metabolic enzymes must be used.

Previous works aimed at identifying protein families associated with the LUCA differ in methodology and conclusions (Becerra et al. 2007; Goldman, Bernhard, et al. 2012). Harris et al. (2003) worked with fully sequenced genomes and used the conserved orthologous groups (COGs) (Tatusov et al. 1997; Koonin 2005; Galperin et al. 2015) as a protein family reference set for analysis. Their approach was strict, in that they focused on genes present in all complete microbial genomes available at the time; 80 COGs were conserved in the analyzed taxa (Harris et al. 2003) (table 1). Fifty of these conserved COGs separated the archaea, bacteria, and eukaryotic domains upon phylogenetic analysis, suggesting presence in the last common ancestor of those domains. A more recent study (Weiss et al. 2016) involved the analysis of de novo clusters of orthologs and focused on protein families which phylogenetically separated archaeal and bacterial taxa in line with recent data suggesting that eukarya are derived from archaea (Raymann et al. 2015; Zaremba-Niedzwiedzka et al. 2017). There, phylogenetic trees which separated the archaea and bacteria by a single branch were compiled, and broad taxonomic distribution (conservation) was not prioritized in the search for LUCA-associated proteins; the presence of an ortholog in two phyla—in addition to phylogenetic separation of the archaea and bacteria—was the set requirement as being a LUCA candidate. Under these criteria, 355 orthologous groups (single split clusters; SSCs) were inferred to be present in the common ancestor of archaea and bacteria (table 1). This latter study was met with some concern (Gogarten and Deamer 2016). Here, we investigated these two previous studies and their contradicting results by reanalyzing original, as well as updated sequence alignments. We also report results from newly developed methods which allow an assessment of interdomain versus intradomain evolutionary distance to test the hypothesis that ancient protein families may exhibit a long interdomain distance relative to intradomain distances (Woese 1998; Forterre 2006; Catchpole and Forterre 2019).

**Table 1.** Table Listing Data Sets Analyzed in This Study.

Name	Number of Protein Families	Number of Domain Separating Families	Underlying Data Set
*SSC*	286,514*	355*	Clusters created by Weiss et al. (2016)
*SSC^COG^*	293	52	SSC composed of corresp. COG sequences
Conserved COGs	80*	50*	COGs, Harris et al. (2003)
Archaeal and bacterial COGs	2,886	661	COGs, Galperin et al. (2015)

Note.—The number of domain separating groups and the corresponding number of domain separating families found in previous studies are marked by * as reported by Weiss et al. (2016) and Harris et al. (2003). *SSC^COG^* is the set of COGs associated with an SSC; these data and those for the total archaeal and bacterial COGs are based on work reported here. Archaeal and bacterial COGs are the set of COGs which include at least one protein sequence from each domain. For details on the construction of the data sets, see Materials and Methods and supplementary sections 2 and 3, and Supplementary Material online.

## Results and Discussion

### Phylogenetic Assessments Are Sensitive to the Number of Sequences Analyzed

**Fig. 1.**
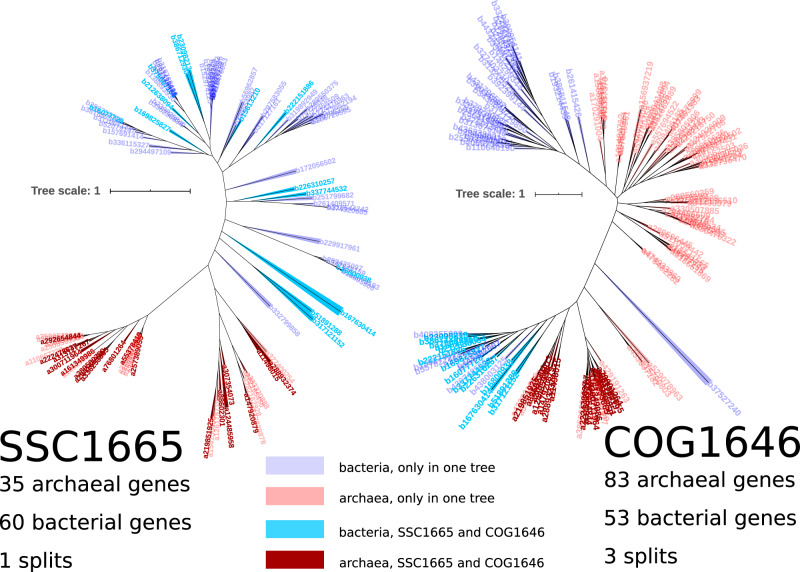
Comparison of tree topologies for two trees corresponding to the same protein family, but which contain different collections of sequences (SSC1665 on the left and COG1646 on the right). Blue colors are bacterial sequences and red colors show archaeal sequences. Sequences with darker color shades appear in both trees; lighter color-shaded labels indicate genes that only appear in a single tree. Leaf labels are gene identifiers .

Figure 1 shows two phylogenetic trees corresponding to portions of one protein family but populated with a different collection of sequences; SSC1665 (Weiss et al. 2016) corresponds to COG1646 (below we refer to COGs which correspond to SSCs as *SSC^COG^*). The SSC shows a single branch (split) separating the archaea and bacteria, but when more sequences are present (as in the COG), three branches separating the domains are observed. As we report below, this loss of archaea:bacteria monophyly in the SSC when more sequences are present is symptomatic of previous work which was used to investigate the protein repertoire of LUCA (Weiss et al. 2016).

Out of the 355 SSCs, 335 families can be assigned to a COG (Weiss et al. 2016). Three of these corresponding COGs lack archaeal sequences, leaving 332 COGs which correspond to the SSC data set. In 35 SSCs, two or more identified protein families were assigned to the same COG, indicating either that these SSC families are portions of larger protein families or that the COG contains paralogous sequences (supplementary additional tables 3 and 4, section 2, and fig. 1, Supplementary Material online). Altogether then, there are 293 unique COGs that can be identified from the original set of 355 SSCs. Of these, only 26 protein families are common with the findings of Harris et al. (2003) (supplementary fig. 1 and table 1, Supplementary Material online).

Phylogenetic reanalysis of the same sequence alignments of Weiss et al. (2016) suggested instability of branch positions in the previous study, since 40 of the clusters reported to have a single branch separating the archaea and bacteria domains exhibited more than one archaea–bacteria split when trees were constructed with IQ-TREE (Nguyen et al. 2015) (supplementary table 5b, Supplementary Material online) (the median interdomain branch support value for trees with more than one separating branch was 0.68; the median interdomain branch support value for the original 355 families constructed with IQ-TREE was 0.9). These results from our reanalysis of the same sequence alignments are consistent with a recent report (Catchpole and Forterre 2019) which did not recover archaea:bacteria monophyly when the sequence alignment of reverse gyrase was reanalyzed. Other studies have also found different results when looking at phylogenetic trees of the same families reported as being in the LUCA (Weiss et al. 2016). For example, the COG of FtsZ was previously highlighted (COG0206) as an example of interdomain horizontal gene transfer (Koonin and Wolf 2008); however, it is found in the list of domain separating LUCA proteins identified in Weiss et al. (2016).

**Fig. 2.**
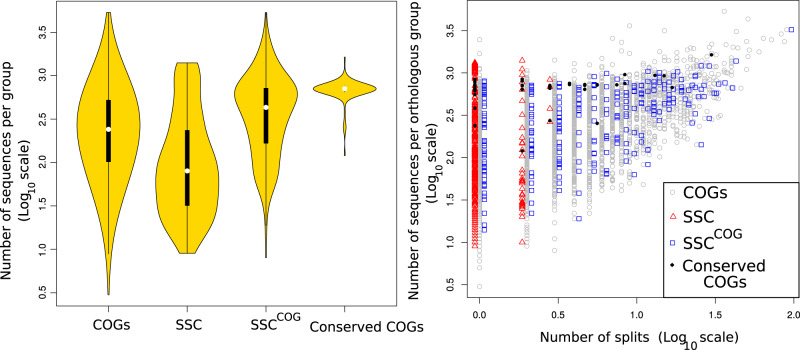
Left: Violin plot depicting the number of sequences per group discussed in the text and table 1. The black bar in the yellow area indicates interquartile ranges. Right: The number of sequences per orthologous group plotted against the number of interdomain branches (splits) found when the sequences are subjected to phylogenetic analysis (log_10_ scales). Expanding *SSCs* (red squares) with the complete set of sequences of the corresponding COGs results in *SSC^COG^* (blue triangles).

Seeking to understand the origins of these conflicting results, we analyzed the number of sequences obtained with different approaches and found that the SSC alignments contain on average less sequences than the corresponding COGs (fig. 2 [left]). Analyzing phylogenies of COGs which correspond to the SSCs (*SSC^COG^*), we found that only 52 trees or ∼18% of the SSC which have a unique corresponding COG show a single branch separating the archaeal and bacterial domains (single split topology; *s *=* *1 and a median branch support at the split nodes of 0.93) (table 1, fig. 2 [right], and supplementary table 1 and additional table 5b, Supplementary Material online). The median branch support of branches separating archaea and bacteria in the trees with more than a single split was 0.82, and the majority of trees with very low branch support (<0.4) at domain separating nodes in the SSC were found with increased branch support values in the *SSC^COG^*, showing that the addition of orthologs improved branch support for some of the protein families (supplementary fig. 15, Supplementary Material online; support values for COG and SSC trees can be found in supplementary fig. 13, Supplementary Material online, and the associated tables). These results show that when the small protein families identified earlier (Weiss et al. 2016) are populated with more sequences, the previously reported monophyly between the archaea and bacteria disappears for most of the families. Including COG-derived trees which exhibit up to three archaea:bacteria branches in their topology, 112 trees (or ∼38% of SSC which have a corresponding COG) match with the reported tree topology of archaea–bacteria separation reported previously (supplementary table 1, Supplementary Material online).

**Fig. 3.**
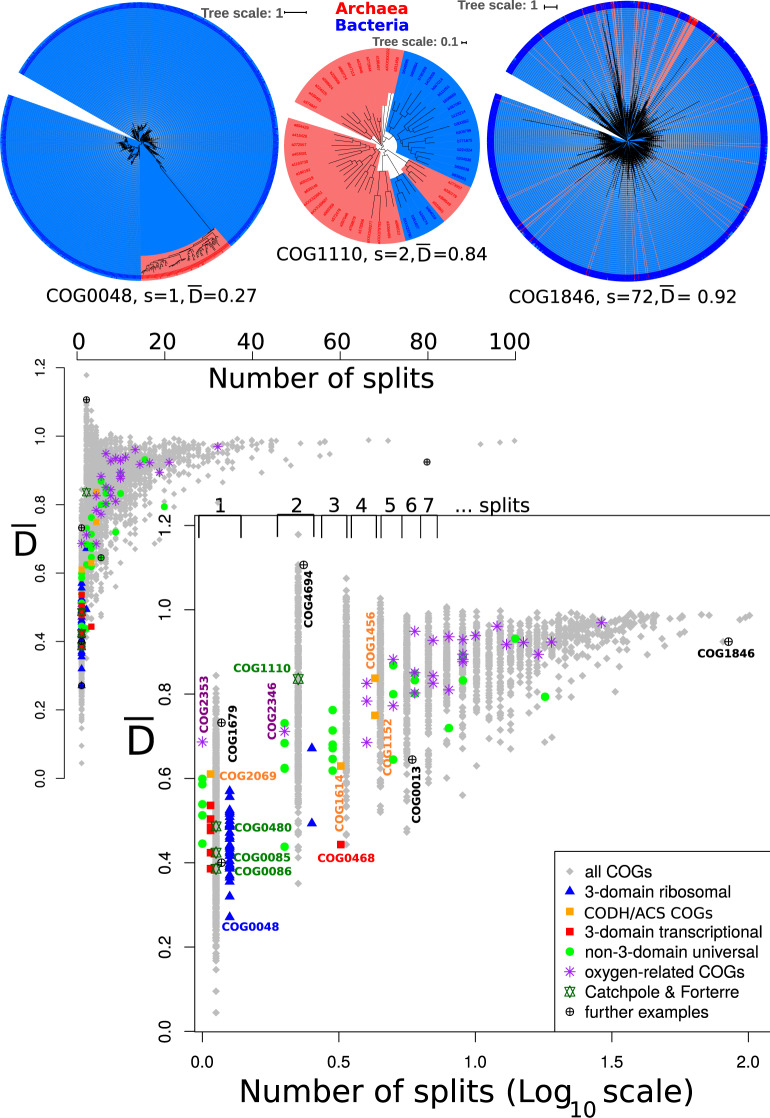
Relationship between the number of archaea:bacteria interdomain branches (splits) and $\bar{D}$ observed in phylogenetic trees drawn from the COGs. Top: Reconstructed trees for COG0048 (ribosomal protein S12), COG1110 (reverse gyrase), and COG1846 (DNA-binding transcriptional regulator, MarR) with corresponding interdomain archaea:bacteria branches (splits) (*s*) and $\bar{D}$ values. The position of these trees is indicated in part B of the figure. The trees are drawn shading archaea in red and bacteria in blue , and the branch lengths are contained within the shaded region. Bottom: Interdomain split values for each COG plotted against $\bar{D}$, where lower $\bar{D}$ values represent phylogenetic trees with smaller average intra- to inter-domain phylogenetic distances. The inset shows the distribution on normal scale, and the log (split) version is shown below. Symbols are slightly shifted to avoid overlays, and the differently shaped and colored symbols indicate subgroups as defined by Harris et al. (2003), Catchpole and Forterre, oxygen related COGs (Liu et al. 2018), CODH/ACS COGs, and further examples as indicated in the legend. Brackets on top of the log-plot summarize regions in the plot that correspond to 1, 2, … splits. Labeled symbols refer to corresponding reconstructed phylogenetic trees shown in (top), in supplementary figure 5 and additional table 2, Supplementary Material online. COG0013 is the alanyl-tRNA synthetase, and COG1679 is a predicted Fe-S cluster binding aconitase.

In contrast, phylogenetic analysis of the 50 conserved three-domain split trees obtained in Harris et al. (2003) with the most recent COG database reveals that 48 trees show a two-domain split (fig. 3). This is remarkable, as the study was conducted 18 years ago and made use of only 34 genomes available at the time. The identified proteins are primarily involved in translation and DNA replication. Sixteen of the 26 conserved COGs of Harris et al. (2003) which overlap with the *SSCs* show a single split between archaea and bacteria upon analysis of the complete set of COGs, whereas 32 conserved COGs which separate the two domains were not identified in the *SSC* (supplementary fig. 1, Supplementary Material online).

### Incorporating Phylogenetic Domain Separation into Tree Analysis

Obtaining accurate groups of orthologs is challenging (e.g., Forslund et al. 2018; Galperin et al. 2019), and as shown above, the analysis of insufficient numbers of sequences can lead to erroneous conclusions. We sought to develop a metric which would aid in overcoming limitations which arise from analyzing incomplete orthologous sets. Long interdomain phylogenetic branches may be indicative of a protein family having been in the LUCA, when the tempo of evolution was rapid, whereas families with shorter branches separating the domains may have originated more recently or exist as examples of recent interdomain gene transfer (Woese 1998; Forterre 2006). Under this theory, phylogenetic trees corresponding to protein families present in the archaea and bacteria decedents of the LUCA are predicted to have long interdomain branches relative to their intradomain branches. Conversely, protein families which evolved after the separation of the archaea and the bacteria are not predicted to show these long domain separating branches. Although this reasoning has previously been applied to a few protein families (Brochier-Armanet and Forterre 2006; Catchpole and Forterre 2019), we here developed a quantitative metric and applied it to a large number of protein families.

$\bar{D}$ describes the ratio of intradomain to interdomain phylogenetic distances found in a tree (Materials and Methods and supplementary section 2, Supplementary Material online), and the three protein families recently analyzed by Catchpole and Forterre (2019) illustrate the utility of this metric. They analyzed the RNA polymerase beta subunit (RpoB COG0085, $\bar{D}=0.42$), elongation factor G (COG0480, $\bar{D}=0.49$), and reverse gyrase (COG1110, $\bar{D}=0.84$) families and noted the difference in branch lengths separating the domains, suggesting that reverse gyrase is not an ancient protein, whereas RpoB and elongation factor G may be. The $\bar{D}$ value quantifies this previous assessment, although a different sequence set (from the COGs) was used here. The reverse gyrase COG (COG1110) contains only a portion of the sequences used in the tree reconstructed in Catchpole and Forterre (2019) and shows only two branches separating the archaea and bacteria domains (fig. 3) (Catchpole and Forterre observed four interdomain archaea:bacteria branches [splits]) with their larger alignment). However, the calculated $\bar{D}$ value from the COG is high, suggestive of a more modern protein family which was subject to interdomain gene transfer (Catchpole and Forterre 2019). Thus, $\bar{D}$ values might be used to supplement phylogenetic inferences based on phylogenetic tree topology, even in the case of incomplete sampling as encountered in this example from the COGs.

Applied to phylogenetic trees drawn from all the COGs, protein families containing a low number of splits between archaea and bacteria groups show variability in $\bar{D}$ values (fig. 3 and supplementary table 1 and additional tables 3 and 4, Supplementary Material online). Families distributed among archaea and bacteria lineages which display one split and low $\bar{D}$ values include some familiar proteins, for example: ribosomal protein S12 (COG0048, $\bar{D}=0.27$, fig. 3), translation elongation factor EF-G (COG0231, $\bar{D}=0.32$), and DNA–RNA polymerase RpoB and C (COG0085, $\bar{D}=0.42$ and COG0086, $\bar{D}=0.39$).

Out of COGs which are represented in at least ten taxa of each domain, 131 of 1751 show a single branch separating archaea and bacteria (supplementary additional table 5a, Supplementary Material online). Among this list are 63 (∼48%) that are within the information functional categories, including various small ribosomal subunits as listed above (supplementary figs. 6–8, Supplementary Material online). Within these protein families exhibiting a single branch separating the archaeal and bacterial domains, variability in $\bar{D}$ exists. Consistent with the finding of variable ages of ribosomal protein components (Kovacs et al. 2017), the ribosomal proteins do not have a coherent $\bar{D}$ value associated between them. For example, ribosomal protein S12 (COG0048) appears to be the most domain separating ($\bar{D}=0.27$), but ribosomal protein L30/L7a (COG1358), which is known to have nonribosomal function (Cho et al. 2010), shows a $\bar{D}$ value of 0.68. A number of protein families with low $\bar{D}$ values overlap with well separated nearly universal trees (Puigbò et al. 2009), indicating that conservation, phylogenetic domain separation, and long interdomain branches coincide for a set of protein families (supplementary fig. 9 and additional table 3, Supplementary Material online).

COGs associated with oxygen metabolism (Liu et al. 2018) all have intra:interdomain phylogenetic distance ratios $\bar{D}>0.59$ and approach $\bar{D}=$1 (fig. 2 and supplementary fig. 10 and table 2, Supplementary Material online). Surprisingly, $\bar{D}$ was also >0.6 for COGs comprising the four subunits of the CODH/ACS enzyme complex homologous within archaea and bacteria, which in contrast to enzymes involved in oxygen metabolism, are thought to be associated with the LUCA (Adam et al. 2018; Inoue et al. 2019), or ancient horizontal gene transfers (Inoue et al. 2019). For example, COG2353 (YceI) (*s *=* *1, $\bar{D}=0.69$) and COG2069 (CdhD) (*s *=* *1, $\bar{D}=0.61$) (see supplementary fig. 10 and table 2, Supplementary Material online, for a full list).

Protein families involved in metabolic processes seem to be less conserved across taxa (Charlebois and Doolittle 2004), more susceptible to lateral gene transfers (Jain et al. 1999), and do not as frequently display long domain separating branches as those in informational categories, for example, COG0636 (the c subunits of the ATP synthase [*s *=* *6, $\bar{D}=0.83$]) and COG1740 and COG0374 ([Ni-Fe] hydrogenase small and large subunits *s *=* *3, $\bar{D}=0.78$ and *s *=* *4, $\bar{D}=0.78$ respectively; see also supplementary figs. 16–19 and additional table 3, Supplementary Material online). Some proteins associated with metabolic functions can however be found with lower $\bar{D}$ values (supplementary additional tables 3 and 6a, Supplementary Material online); for example, the Fe-S oxidoreductase COG1625 (*s *=* *2, $\bar{D}=0.47$), the beta subunit of Coenzyme F420-reducing hydrogenase COG1035 (*s *=* *1, $\bar{D}=0.55$), and triosephosphate isomerase COG0149 (*s *=* *2, $\bar{D}=0.43$) may suggest ancient electron transfer and sugar metabolism. COG1229 (the formylmethanofuran dehydrogenase subunit A *s *=* *2, $\bar{D}=0.62$) might also be considered as ancient, but as the number of interdomain split values increases, strong conclusions of the physiology of the LUCA are precluded in the absence of more detailed phylogenetic analysis.

### Phylogenetic Topology, and Domain Separation Is Nonrandom for a Set of Protein Families

The majority of COGs are composed out of ∼10% and ∼90% of proteins from each domain (supplementary fig. 11, Supplementary Material online). In a permutation analysis, we took the topology of trees derived from the COGs and shuffled archaea and bacteria in different proportions to create trees of random distributions of archaea and bacteria mapped onto the original trees derived from the COGs. Trees drawn from biological data sets are dramatically different from these random sampling iterations. Only trees which are derived from a low number of sequences (<10 genes per group) showed a single split, and the $\bar{D}$ values do not decrease below 0.51 for these single split trees (supplementary fig. 12, Supplementary Material online). For simulated trees with at least ten genes per group, the minimal number of interdomain branches is 5, which contrasts to the set of COGs where 131 single split trees can be found with at least ten each of archaea and bacteria (supplementary section 4 and additional table 6b and Material and Methods, Supplementary Material online). The observation that domain separating single branches can be obtained by chance in the permutation analysis may be similar to some of the results of Weiss et al. (2016), where 184 protein families of the 355 identified have <10 sequence representatives of archaea or bacteria (supplementary additional table 4, Supplementary Material online). False positives happen by chance more often when there are less sequences (supplementary fig. 12, Supplementary Material online).

### Diversity of Evolutionary Mode and History among Protein Families

It has been suggested that proteins present in the LUCA would have a long interdomain phylogenetic branch, reflecting high evolutionary rates before what Woese referred to as crystallization of the domains (Woese 1998; Brochier-Armanet and Forterre 2006; Forterre 2006). This does appear to be reflected in some LUCA protein families (Catchpole and Forterre 2019), and our analysis of $\bar{D}$ values is a broad test of this hypothesis.

The majority of protein families have intradomain branch lengths that are less than or equal to the interdomain distance, and information categories of proteins are enriched in trees with long interdomain branches. That branch lengths between the archaea and bacteria domains are generally longer than within domain branch lengths is consistent with a hypothesis of a high tempo of evolution prior to the separation of the domains, but the diversity of branch length ratios between protein families is suggestive of unique evolutionary pressures and histories between families. This may be especially relevant considering the diversity of $\bar{D}$ values observed for proteins which are likely to have been in the LUCA (most prominently the ribosomal proteins).

Only a few protein families show $\bar{D}$ values >1. These protein families contain one, or a very small number of sequences from one of the domains (e.g., COG4694, annotated as the tRNase RloC has only two archaeal sequences, which each resulting in a archaea:bacteria branch). It could be that these archaeal sequences do not belong in the cluster or are recent interdomain gene transfers.

Although $\bar{D}$ values supplement phylogenetic inference by introducing a distance metric, they do not themselves provide an independent criterion for accessing if a protein family was in the LUCA. It is possible that some proteins may have been in the LUCA but do not show long interdomain branches, and some protein families which were likely in the LUCA simply do not have a single branch separating the domains (Hilario and Gogarten 1993; Wolf et al. 1999; Gogarten and Deamer 2016). In many cases, simply counting the number of domain separating branches in a phylogenetic tree is insufficient to account for the realities of LGT and loss. Instead, careful phylogenetic analysis is needed to infer protein ancestry, as for example in the case of the CODH/ACS complex (e.g., Adam et al. 2018; Inoue et al. 2019).

From the perspective of very early life, it could be that some LUCA proteins might easily undergo interdomain gene transfer, which would blur the ability to recognize them as ancient by a low number of splits. Indeed, Woese’s theory of genetic annealing postulated both mutational rate and lateral gene transfer as components of what may have been a high “temperature” in predomain evolution (Woese 1998). Such easily transferred proteins with “erased” signals of antiquity could be advantageous if early communities relied on horizontal, rather than vertical inheritance (as, e.g., in the stage of a progenote [Woese and Fox 1977; Woese 1998]).

## Prospectus

### Outstanding Orthology Problem

Various approaches exist to detect sets of orthologous sequences, which remains an ongoing challenge (Lechner et al. 2014; Forslund et al. 2018). In our analysis of $\bar{D}$ and the number of interdomain splits, both missing orthologs, and the addition of paralogs in the COGs could affect our results. The COGs are a well-known data set for example (Harris et al. 2003; Charlebois and Doolittle 2004; Puigbò et al. 2009; Goldman, Baross, et al. 2012) created by defining orthology based on sequence comparison and function annotation. This is in line with the orthology conjecture, which states that the most closely related sequences will have the most closely related function (Koonin 2005; Forslund et al. 2018). Incomplete genome annotation, inaccurate function annotation, and a yet incomplete understanding of the cellular environments where proteins function (Nehrt et al. 2011) make this definition subject for debate. Community efforts to create accurate sets of orthologs (Altenhoff et al. 2016) with increased microbial representation will be critical for future work.

Annotation issues can be corrected by merging bioinformatics with the granularity of biochemistry, but these still confuse analyses aimed at understanding evolution. For example, the putative phosphate acetyltransferase (Pta) sequences found in Weiss et al. (2016) lack catalytic residues (Lawrence et al. 2006) and align poorly with the *Escherichia coli* and *Methanosarcina thermophila* proteins, meaning that the identified protein is likely different and cannot function as imagined in that report in early energy conservation (i.e., conversion).

### Concluding Remarks

Our work furnishes a new variable for the assessment of protein family evolution which compliments previous approaches based on conserved presence and phylogenetic topology. Using phylogenetic tree–based approaches of the type used here, only limited information can be gained about the LUCA, leaving specific details on physiology largely speculative. Analysis of proteins such as the reverse gyrase, hydrogenase, and nitrogenase discussed here and elsewhere (Boyd, Anbar, et al. 2011; Boyd, Hamilton, et al. 2011; Catchpole and Forterre 2019) does not support the conclusion of a thermophilic, nitrogen fixing and hydrogen utilizing LUCA (Weiss et al. 2016).

The evolutionary signal of proteins involved in cellular informational processes appears different than those involved in metabolism, and it could be that the modularity of energy metabolism is in part responsible for an erosion of signal in this latter category. Many of the protein families involved in transcription and protein synthesis do not appear to display interdomain modularity (consistent with the complexity hypothesis; Jain et al. 1999). Their low split values and broad taxonomic distribution are suggestive of their presence in the LUCA, and their small intra:interdomain phylogenetic distance ratios may reflect high early evolutionary temperatures.

It may be beneficial to integrate protein structure information to better estimate phylogenetic distances. In addition, orthologous groups identified by new methods can be usefully referenced and compared with results from other studies. For example, the nearly universal trees are a set of conserved protein families with variable degrees of domain separation (Puigbò et al. 2009, 2010). Going further, employing recent phylogenetic methods such as reconciling gene trees with species trees (Altenhoff and Dessimoz 2012; Hellmuth 2017) may aid in overcoming problems associated with limited gene distribution among taxa (Charlebois and Doolittle 2004); however, this is dependent on the availability of reliable species trees. In an effort to integrate molecular data into an Earth history context, geochemical data can give further clues about the environmental conditions on early Earth, allowing for phylogenetic–geochemical calibrations to be made (Wolfe and Fournier 2018; Shih et al. 2017). Altogether, analyses integrating data from multiple dimensions might refine the concept of, and the evolutionary scenario suggested by the statistical tree of life (Puigbò et al. 2009; O’Malley and Koonin 2011; Doolittle and Brunet 2016).

The physiology of the LUCA remains largely unconstrained. A remaining challenge is to understand the evolutionary distance, and molecular differences between the LUCA and the forms of life which came before it (Gogarten and Deamer 2016; Cornish-Bowden and Cárdenas 2017).

## Materials and Methods

In order to compare different approaches, we downloaded multiple sequence alignments (MSAs) for COGs (https://www.ncbi.nlm.nih.gov/COG, last accessed April 15, 2020) (Tatusov et al. 1997) and collected corresponding COGs given in Harris et al. (2003) and Catchpole and Forterre (2019). The phylogenetic trees and alignments used to obtain conclusions in Weiss et al. (2016) were not published in that study and were instead obtained from author contact on the now defunct pubmedcommons site (ftp://ftp.ncbi.nlm.nih.gov/pubmed/pubmedcommons). After downloading all trees and alignments from the former study, they were subsequently used in our analyses. Corresponding gene families in the COG data set were given for 335 of the 355 clusters identified in Weiss et al. (2016). We used FastTree (Price et al. 2010) with default parameters and IQ-TREE (Nguyen et al. 2015) with -bb 1000 for bootstrap support and -m JTT specifying the evolutionary model, to reconstruct trees based on the MSAs of Weiss et al. (2016) and on MSAs for the data set of all COGs. The analysis presented in the main text is based on IQ-TREE results, but we also employed FastTree separately and obtained similar results. A short comparison between FastTree and IQ-TREE results can be found in the supplementary section 3, Supplementary Material online. The study of Weiss et al. (2016) used RaxML (Stamatakis 2014) to build phylogenetic trees, however, we obtained almost the same results (table 1). We only include COGs in the study that contain archaeal as well as bacterial sequences. This is not the case for COG0050 (the current COG set does not contain archaeal sequences), which is contained in the data set by Harris et al. (2003), as they additionally included eukaryotic sequences. Therefore, we only include 79 gene families from the study by Harris et al. (2003). Further information on the data sets can be found in supplementary section 2, Supplementary Material online. In order to obtain one-to-one orthologs sets for the COGs, obvious paralogous sequences from the same species were removed.

After constructing trees for each COG, we calculated the number of archaea:bacteria branches (splits *s*) needed to separate archaeal (A) and bacterial (B) genes. This was done with a modified version (github.com/bsarah/treeSplits) of the Fitch algorithm (Fitch 1971). Given a tree, we detect nodes in the tree that represent the lowest common ancestor (lca) of a (possibly maximal) set of archaeal or bacterial species. The trees are binary, thus the parent node *p* of this lca will have two children nodes, each one spanning a subtree of a different domain. In order to calculate support values for split nodes, we take the support value at *p*. In case of several splits in the tree, we calculate the average support value. Trees were visualized using iTOL (Letunic and Bork 2007, 2016).

Distances between sequences can be calculated by summing up branch lengths on the path between pairs of leaves of the tree. As shown in figure 3, a tree may show a single split when at least one of the groups is closely connected, thus average pairwise distances are relatively small. Therefore, we calculated the mean phylogenetic pairwise distances between leaves for intragroup genes (between only archaeal and only bacterial sequences) and intergroup distances, that is, the distances between archaeal and bacterial proteins. The following formulas show how calculations were conducted. Here, *A* is the set of archaeal and *B* the set of bacterial genes in a tree, with sizes *n* and *m*, respectively. The function $d_{t}(a_{i},a_{j})$ calculates the distance in the tree *t* between archaeal genes *a_i_* and *a_j_* and analogously for $d_{t}(b_{i},b_{j})$. Then, ${\bar{d}}_{AA}(t)$ and ${\bar{d}}_{BB}(t)$ are the mean pairwise distances between archaeal (bacterial) species in tree *t*:

$${\bar{d}}_{AA}(t)=\frac{\sum_{i,j=1}^{n}d_{t}(a_{i},a_{j})}{n\cdot (n-1)},\;{\bar{d}}_{BB}(t)=\frac{\sum_{i,j=1}^{m}d_{t}(b_{i},b_{j})}{m\cdot (m-1)}.$$

The same can be done in order to calculate distances between genes from different groups, thus $d_{AB}(t)$ gives the mean pairwise distance between intergroup genes for tree *t*:

$${\bar{d}}_{AB}(t)=\frac{\sum_{i=1}^{n}\sum_{j=1}^{m}d_{t}(a_{i},b_{j})}{n\cdot m}.$$

For each tree *t*, there is a set of genes for archaea and a set of genes for bacteria. We calculate the distance between all possible pairs of archaeal gene *a* and bacterial gene *b* by summing up over all archaeal and bacterial genes. As the value is dependent on the tree *t*, we indicate this by writing *d_t_*. These distances can now be used to calculate the ratio of how closely related genes in one group (intragroup) are in comparison to intergroup distances:

$$\bar{D}=\frac{1/2\cdot ({\bar{d}}_{AA}+{\bar{d}}_{BB})}{{\bar{d}}_{AB}}.$$

A further possibility is to only consider the group of genes that has closer mutual relationships replacing the mean value by the minimum:

$$D=\frac{\min({\bar{d}}_{AA},{\bar{d}}_{BB})}{{\bar{d}}_{AB}}.$$

Values for $\bar{D}$ are at least equal or larger than the corresponding *D* value. Values for $\bar{D}$ are plotted in figure 3 and included in further supplementary figures and tables, Supplementary Material online. Values for *D* and $\bar{D}$ are also denoted as Dmin and Dav in the supplementary tables, Supplementary Material online, respectively.

In order to have a randomized reference set of trees, domain identifiers marked at the leaves (*A* for archaea or *B* for bacteria) were shuffled on trees built with FastTree from the full set of COG alignments which contain archaeal and bacterial sequences. Thus, topology and size were kept and for each tree, we randomly set the labels to *A* or *B*. This exercise was performed with three varied proportions of archaea *A* and bacteria *B* in the trees: 1) 30% *A* and 70% *B*, 2) 50% *A* and 50% *B*, and 3) 90% *A* and 10% *B*. For each of the trees in the randomized data sets, the number of splits and values for $\bar{D}$ were calculated. Distribution of values for splits *s* and $\bar{D}$ compared with COGs are plotted and shown in supplementary figure 12, Supplementary Material online.

## Data Availability

Data sets used in this study including reconstructed phylogenetic trees and randomized trees are available at www.bioinf.uni-leipzig.de/supplements/19-004.

## Supplementary Material

msaa089_Supplementary_Data
