# Supplementary material for: A New Analysis of Archaea–Bacteria Domain Separation: Variable Phylogenetic Distance and the Tempo of Early Evolution
Source: Mol Biol Evol. 2020 Apr 21;37(8):2332–40. doi: 10.1093/molbev/msaa089 (PMC7403611; doi:10.1093/molbev/msaa089)
Supplement: msaa089_Supplementary_Data [file msaa089_supplementary_data.zip › msaa089-suppl_data/BerkemerMBE_final_supplemental.pdf]

Supplemental Data
A new analysis of archaea-bacteria domain separation: variable phylogenetic distance and the tempo of early evolution

Sarah J. Berkemer and Shawn E. McGlynn

1 Supplemental Data

The supplement includes the following figures and tables:

1. Figure 1 depicting overlaps and numbers of sequences between distinct sets of COGs.

2. Figure 2: Number of archaea:bacteria interdomain branches (splits) plotted against the number of sequences in each orthologous group in normal scale and log_{10} scale for four data sets: data set of COGs [9], SSC by Weiss et al. [10], SSC_{COG}, and conserved COGs by Harris et al. [3].

3. Figure 3, plotting number of archaea:bacteria interdomain branches (splits) against (A) D and (B) $\overline{D}$ for COGs, SSC and SSC^{COG}. D is the ratio of the minimum of the average pairwise distances within archaea or bacteria, to the average pairwise distance between domains. $\overline{D}$ is the average of pairwise distances within both archaeal and bacterial domains, to the average pairwise distance between domains.

4. Figure 4, plotting number of archaea:bacteria interdomain branches (splits) against D for all COGs, COGs analysed by Harris et al and COGs analysed by Catchpole & Forterre.

5. Figure 5: Phylogenetic trees for COG0048 (Ribosomal protein S12), COG1110 (Reverse Gyrase) and COG1846 (DNA-bind. transcript. regulator, MarR fam.) with archaeal clades in red and bacterial clades in blue.

6. Figure 6: Frequencies for functional categories for the set of COGs, in total 4631 COGs.

7. Figure 7: Frequencies for functional categories for the set of COGs with at least 10 archaeal and 10 bacterial sequences, in total 1751 COGs.

8. Figure 8: Frequencies for functional categories for the set of COGs with at least 10 archaeal and 10 bacterial sequences and a single split tree topology, in total 131 COGs.

9. Figure 9: Values for D and $\overline{D}$ plotted against number of splits in log_{10} scale for the data set of COGs (gray) and the NUTs data set [8] corresponding to separation scores. A separation score of 1 (red) indicates trees which separate the domains, whereas a lower separation score (blue) indicates interdomain mixing of sequences in the phylogenetic tree. Values for D, $\overline{D}$ and splits are calculated for reconstructed phylogenetic trees based on the COG data set.

10. Figure 10 showing number of archaea:bacteria interdomain branches (splits) plotted against D and $\overline{D}$ for the data sets of CODH/ACS COGs [1, 4] and oxygen related COGs [5]

11. Figure 11 showing distribution and frequencies of COGs with varying percentages of archaeal and bacterial species.

12. Figure 12 comparing number of archaea:bacteria interdomain branches (splits) in log_{10} scale to $\overline{D}$ (blue) and the number of sequences per group in log_{10} scale (gray) for the set of COGs and randomized trees including all trees (top) and trees with at least 10 leaves of each label, a and b (bottom).

13. Figure 13 showing distribution of support values for `IQTREE` and `FastTree`

14. Figure 14 comparing split values obtained using `IQTREE` and `FastTree` for the reconstruction of phylogenetic trees based on the COGs

15. Figure 15 comparing support and split values for COGs, SSCs, and SSC^{COG} when including all groups in the analysis or only including groups containing at least 10 archaeal and 10 bacterial sequences.

16. Figure 16 showing distribution of $\overline{D}$ values in the functional categories for all COGs with archaeal and bacterial proteins.

17. Figure 17 showing distribution of $\overline{D}$ values in the functional categories for all COGs including at least 10 archaeal and 10 bacterial proteins.

18. Figure 4 showing distribution of $\overline{D}$ values in the functional categories for all COGs with a single split.

19. Figure 19 showing distribution of $\overline{D}$ values in the functional categories for all COGs including at least 10 archaeal and 10 bacterial proteins separated by a single split.

20. Table 1 listing COGs with single split tree topology for COGs.

21. Table 2 listing COGs with archaea:bacteria interdomain branches (splits), number of sequences, functional category, $\overline{D}$ and annotation for oxygen related COGs [5], CODH/ACS COGs [1, 4] and COGs analysed by Catchpole & Forterre [2].

22. Additional Table 3 including data for COGs and corresponding reconstructed phylogenetic trees with number of splits and pairwise intra- and interdomain distances. This table also includes overlaps with data sets by Harris et al and Weiss et al, values for D and $\overline{D}$ and overlaps with the NUTs data set. The table additionally includes values for splits, support, D and $\overline{D}$ for results obtained using `IQTREE` [6] and `FastTree` [7].

23. Additional Table 4 including data for clusters created by Weiss et al. [10] and corresponding reconstructed phylogenetic trees. The table additionally includes values for splits, support, D and $\overline{D}$ for results obtained using `IQTREE` [6] and `FastTree` [7].

24. Additional Tables 5a and 5b listing COGs (5a) and SSCs (5b) showing a single split topology after reconstruction of phylogenetic trees using `IQTREE` [6]. These tables are also included in additional tables 3 and 4.

25. Additional Table 6a, listing COGs with at least 10 sequences per domain, in total 1751 COGs and additional Table 6b listing COGs with at least 10 sequences per domain and single split topology, in total 131 COGs. These two tables are ascendingly sorted by $\overline{D}$ values. The data collected in these tables is also included in additional tables 3 and 4.

2 Data Sets

Our reconstruction of phylogenetic trees was based on three different data sets: COGs by Tatusov et al. [9], conserved domain separating COGs by Harris et al. [3], and single split clusters (SSC) created by Weiss et al. [10]. In their data sets, Harris et al. [3] list 80 COGs with 50 having a single (3-domain) split topology and Weiss et al. [10] discovered a set of 355 clusters that separated archaeal and bacterial species.

The reconstruction of phylogenetic trees based on the same multiple sequence alignments of Weiss et al. [10] (SSC) resulted in 315 trees with a single split, 36 with two splits and 4 trees where three splits are needed to separate archaea and bacteria (min 9 sequences, median 80, mean 230 sequences). The difference between these results and the previous appear to result from differences in phylogenetic tree building algorithm, and may indicate instability in the branch positions. Out of the 355 groups identified by Weiss et al. [10], 335 SSC corresponded to a COG, which results in 293 distinct COGs as several SSC were assigned to the same COG. We reconstructed phylogenetic trees corresponding to the groups of Harris et al. [3] as well as these 293 COGs and call this set SSC^{COG}. The single split COGs in the set of SSC^{COG} are listed in Table 1.

The following tables list sizes of data sets used in this study:

Data sets based on other publications:

	total	single split
SSC	286514	355
Harris et al.	80	50

Data sets with phylogenetic trees reconstructed using IQTREE [6]:

	total	single split	numbers of proteins
SSC(IQ)	355	315	min 10, mean 242, median 84
SSC(IQ and COG)	335	296	min 10, mean 238, median 83
Harris et al.(IQ)	80	48	min 120, mean 698, median 708
SSC^{COG}	293	52	min 8, mean 568, median 431
mixed COGs	2886	661	min 3, mean 348, median 241

The table above contains data sets where phylogenetic trees were reconstructed using `IQTREE`. The reconstruction of SSC(IQ) is based in the multiple sequence alignments provided by Weiss et al. The set SSC(IQ and COG) only takes into account SSC with a corresponding COG, and are populated with the original sequences of Weiss et al. The other trees are reconstructed based on the corresponding set of COGs. The term 'mixed COGs' corresponds to the set of COGs which contain at least one archaeal and one bacterial protein sequence. The set of SSC^{COG} is built based on the set of 293 distinct COGs that were referred to corresponding to the set of SSC.

3 Reconstruction of Phylogenetic Trees

Phyogenetic trees were reconstructed based on the multiple sequence alignments (MSAs) for the set of COGs[1] [9] and the MSAs of [10] obtained from author contact. We used `FastTree` [7] and `IQTREE` [6].
`FastTree` was used with default parameters. For `IQTREE` we used the parameters `-bb 1000` for bootstrap support and `-m JTT` specifying the evolutionary model. For each tree, we calculated the average support value at the split node(s), separating archaeal and bacterial proteins in the tree.
Figure 13 shows the distribution of support values for COGs and SSCs for trees reconstructed with `FastTree` (median: 0.73) and `IQTREE` (median: 0.81). For `IQTREE` we obtained on average higher support values, thus our results are based on trees built using `IQTREE`. We additionally calculated the distribution of support values for groups of proteins that include at least 10 archaeal and 10 bacterial proteins to avoid low support values due to very low numbers of

[1] `https://www.ncbi.nlm.nih.gov/COG`

protein sequences.
Figure 14 compares split values obtained using `IQTREE` and `FastTree` for the reconstruction of phylogenetic trees based on the COGs. The red line shows the diagonal with a slope of 1. Different results for splits were obtained if the corresponding symbol in the plot is located apart from the diagonal line. For most of the COGs (1886 of 2886), `IQTREE` and `FastTree` obtained the same number of splits. For 690 COGs, split values differ by 1 and in 195 cases, calculated numbers of splits show a difference of 2. The remaining 115 COGs showed differences in split numbers between 3 and 8.

4 Randomized Trees

Phylogenetic trees based on the COGs were constructed using `FastTree` [7]. Leaves were labeled by taxon identifier and marked A or B to indicate the species belonging to the domain of archaea or bacteria. In order to have a randomized reference set of trees, domain identifier were shuffled. Thus, tree topology and size were kept and for each leaf, we randomly chose to set the label to A or B. This resulted in three data sets with the following percentages of A and B labels:

- 30% A and 70% B,
- 50% A and 50% B,
- 90% A and 10% B.

For each of the trees in the randomized data sets, the number of splits and values for D and $\overline{D}$ were calculated. Figure 12 shows distribution of the number of splits, values for $\overline{D}$ and size of groups for the three randomized data sets in comparison to the set of COGs.

The set of COGs can be clearly distinguished from the randomized sets of trees based on the distribution of symbols. It can be seen that $\overline{D}$ values are mainly distributed between 0 and 1 for COGs whereas the distribution is significantly shifted upwards for the randomized trees. The plots showing the group size indicate a clear correlation between number of splits and group size for randomized trees which is not the case for COGs where trees of various sizes are present within the range of low numbers of splits.

As depicted in Figure 12 (bottom), there are no trees with less than 5 splits for the randomized trees. This clearly shows that a low number of splits highly correlates with a low number of sequences per group. For the COGs with at least 10 protein sequences in each domain, we get a median of 7 splits. For the randomized trees, median values of splits are 29 (90% A, 10% B), 55 (50% A, 50% B) and 47 (30% A, 70% B).

The plots in Figure 12 clearly show the difference between randomized trees and COGs. The distribution of $\overline{D}$ values for the COGs shows that intra-group distances are mostly smaller than inter-group distances which is not the case for the randomized trees. The plots also underline the importance of the correlation

between the number of protein sequences per domain and the number of splits. The permutation trees suggest that it is more probable to get randomized tree topologies with low number of splits if the trees are small or the proportion of the amount of proteins within each domain is unbalanced. This leads to the assumption that COGs with a higher number of proteins (in both domains) provide more reliable data than smaller gene clusters. Of course, a well-distributed set of proteins over the tree of life is fundamental to evolutionary inference.

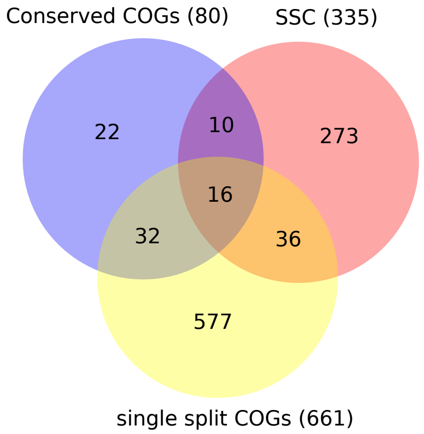

Figure 1: Overlapping COGs between the complete data sets by Harris et al [3] (Conserved COGs, blue), Weiss et al [10] (SSC, red), and the set of COGs which separate the archaea and bacteria (single split COGs) when analyzed by IQTree, based on the COG data set by Tatusov et al [9] (yellow). Harris et al. identified 50 conserved COGS which showed a 3 domain separation topology. 48 of these are retained as separating the two domains using the latest release of the COGs. The set of Weiss et al. (SSC) only contains groups with a corresponding COG. Out of the 355 SSCs, 335 families can be assigned to a COG 3 of these corresponding COGS lack archaeal sequences, leaving 332 COGS which correspond to the SSC data set. In 35 SSCs, two or more identified protein families were assigned to the same COG.

References

[1] P. S. Adam, G. Borrel, and S. Gribaldo. Evolutionary history of carbon monoxide dehydrogenase/acetyl-CoA synthase, one of the oldest enzymatic complexes. *Proceedings of the National Academy of Sciences*, 115(6):E1166–E1173, 2018.

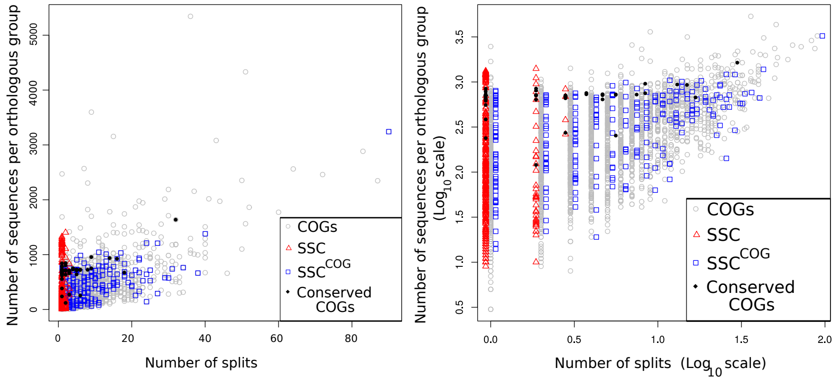

Figure 2: Number of splits plotted against number of sequences in orthologous groups in normal scale (left) and log_{10} scale (right) for four data sets: data set of COGs [9], SSC [10], SSC_{COG} and conserved COGs by Harris et al. [3].

[2] R. Catchpole and P. Forterre. The evolution of reverse gyrase suggests a non-hyperthermophilic last universal common ancestor. *Molecular Biology and Evolution*, 2019.

[3] J. K. Harris, S. T. Kelley, G. B. Spiegelman, and N. R. Pace. The genetic core of the universal ancestor. *Genome research*, 13(3):407–412, 2003.

[4] M. Inoue, I. Nakamoto, K. Omae, T. Oguro, H. Ogata, T. Yoshida, and Y. Sako. Structural and phylogenetic diversity of anaerobic carbon-monoxide dehydrogenases. *Frontiers in Microbiology*, 9, 2019.

[5] S. Liu, M.-Z. Du, Q.-F. Wen, J. Kang, C. Dong, L. Xiong, J. Huang, and F.-B. Guo. Comprehensive exploration of the enzymes catalysing oxygen-involved reactions and COGs relevant to bacterial oxygen utilization. *Environmental Microbiology*, 20(10):3836–3850, 2018.

[6] L.-T. Nguyen, H. A. Schmidt, A. von Haeseler, and B. Q. Minh. IQ-TREE: a fast and effective stochastic algorithm for estimating maximum-likelihood phylogenies. *Molecular biology and evolution*, 32(1):268–274, 2014.

[7] M. N. Price, P. S. Dehal, and A. P. Arkin. Fasttree 2–approximately maximum-likelihood trees for large alignments. *PloS one*, 5(3):e9490, 2010.

[8] P. Puigbo, Y. I. Wolf, and E. V. Koonin. Search for a 'tree of life' in the thicket of the phylogenetic forest. *Journal of biology*, 8(6):59, 2009.

[9] R. L. Tatusov, E. V. Koonin, and D. J. Lipman. A genomic perspective on protein families. *Science*, 278(5338):631–637, 1997.

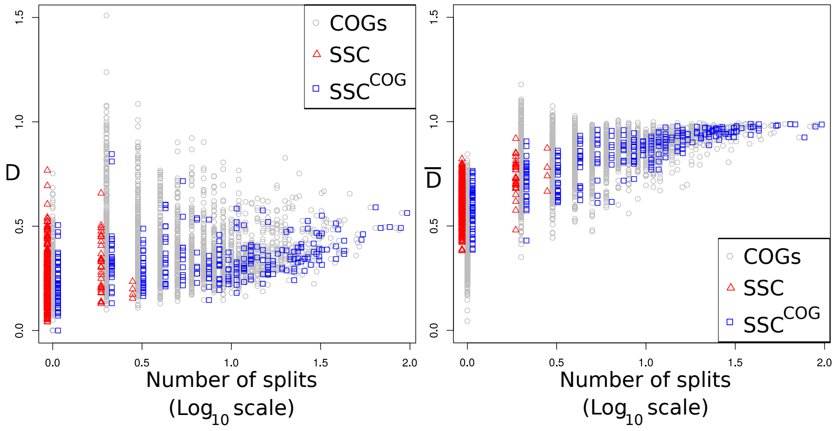

Figure 3: D plotted against number of splits in log_{10} scale, for the data sets of COG (gray), SSC (red) and SSC^{COG} (blue) (left). $\overline{D}$ plotted against number of splits in log_{10} scale, for the data sets of COG (gray), SSC (red) and SSC^{COG} (blue) (right).

[10] M. C. Weiss, F. L. Sousa, N. Mrnjavac, S. Neukirchen, M. Roettger, S. Nelson-Sathi, and W. F. Martin. The physiology and habitat of the last universal common ancestor. *Nature Microbiology*, 1(9):16116, 2016.

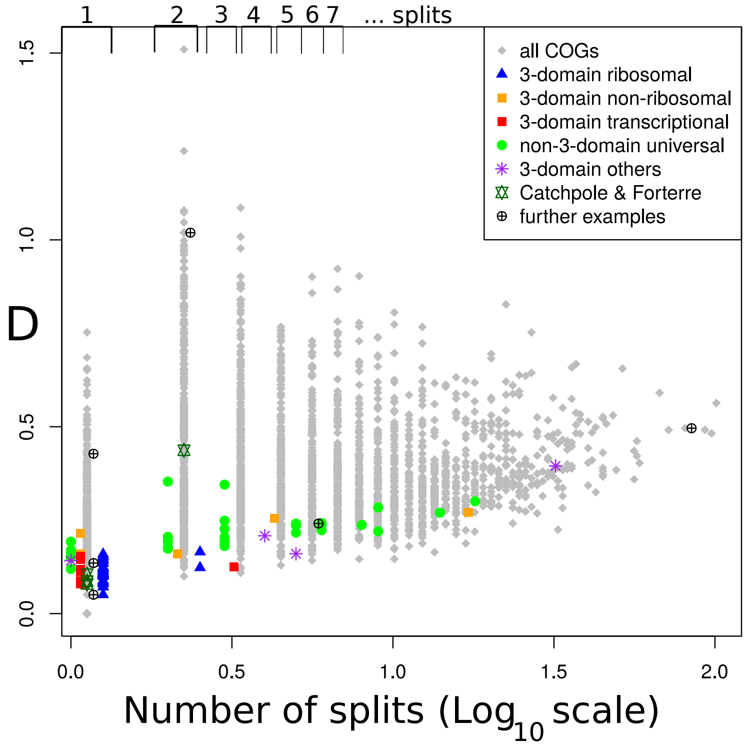

Figure 4: Values for D against number of splits in log10 scale. The symbols are slightly shifted to avoid overlays. Gray symbols represent all COGs, colorful symbols show groups defined by Harris et al (group 1: blue, group 2: orange, group 3: red, group 5: green, groups 4 & 6: purple). COGs corresponding to protein families in Catchpole & Forterre are marked in dark green. Further examples displayed in this work are marked in black.

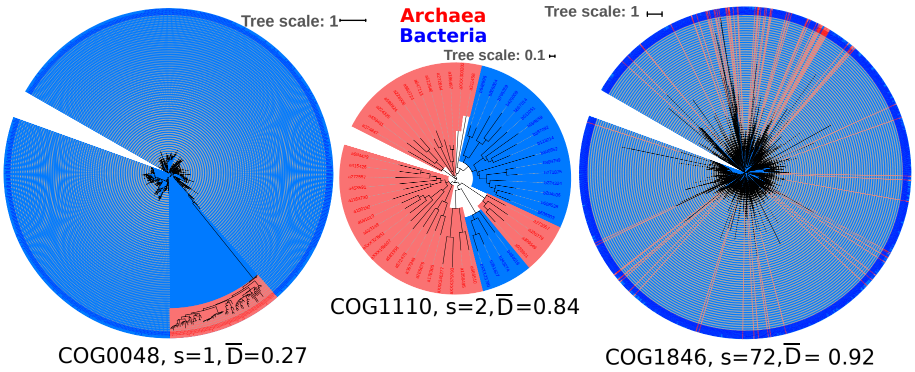

Figure 5: Phylogenetic trees for COG0048 (Ribosomal protein S12), COG1110 (Reverse Gyrase) and COG1846 (DNA-bind. transcript. regulator, MarR fam.) with archaeal clades in red and bacterial clades in blue.

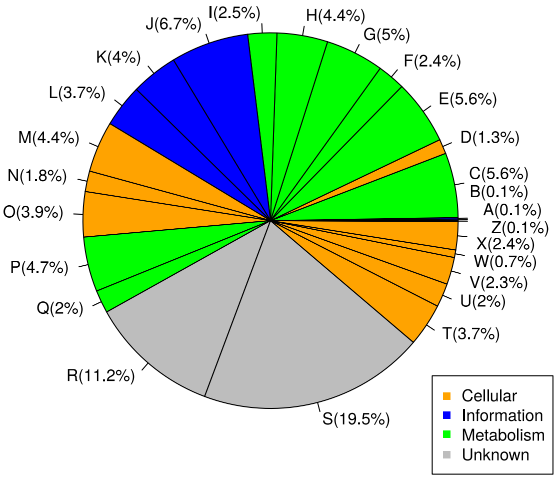

Figure 6: Frequencies for functional categories for the set of COGs, in total 4631 COGs.

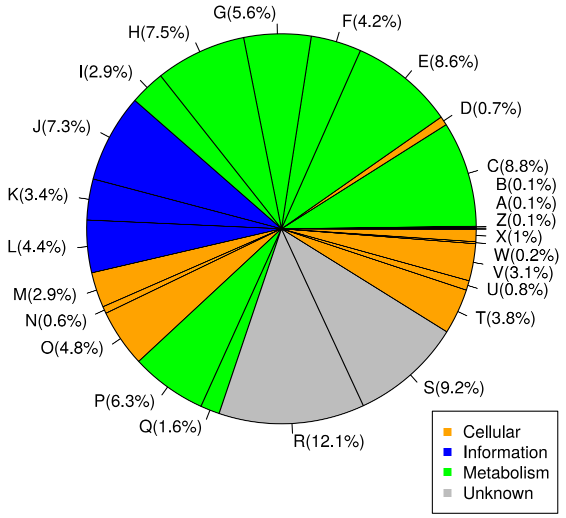

Figure 7: Frequencies for functional categories for the set of COGs with at least 10 archaeal and 10 bacterial sequences, in total 1751 COGs.

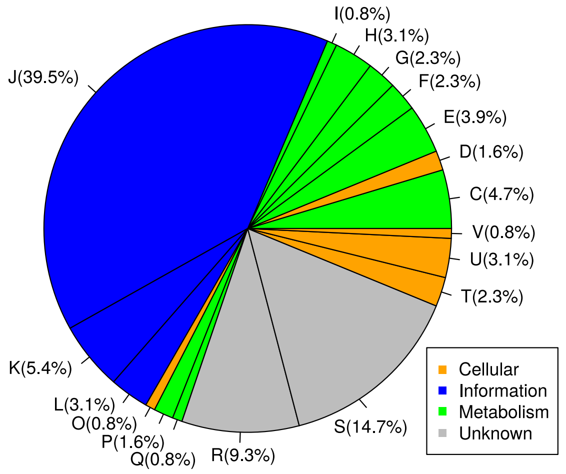

Figure 8: Frequencies for functional categories for the set of COGs with at least 10 archaeal and 10 bacterial sequences and a single split tree topology, in total 131 COGs.

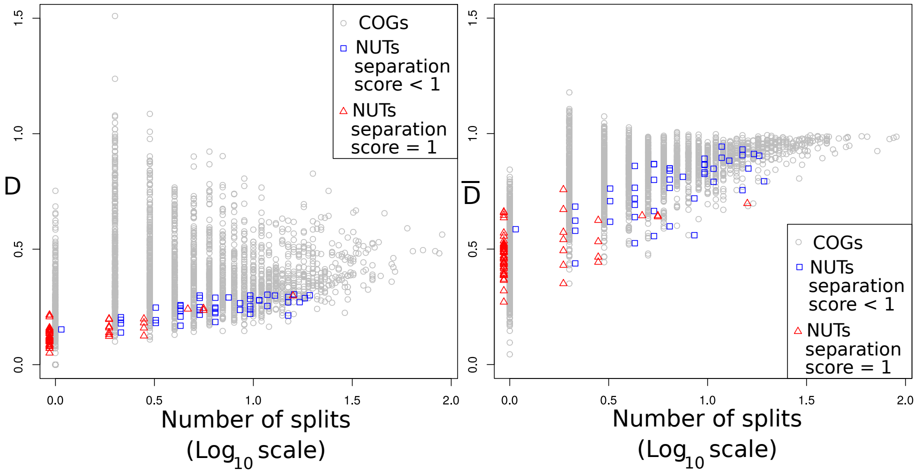

Figure 9: Values for D (left) and $\overline{D}$ (right) plotted against number of splits in log_{10} scale for the data set of COGs (gray) and the NUTs data set [8] corresponding to separation scores. A separation score of 1 (red) indicates trees which perfectly separate the archaea and bacteria, whereas a lower separation score (blue) indicates interdomain mixing of sequences in the phylogenetic tree. Values for D, $\overline{D}$ and splits are calculated for reconstructed phylogenetic trees based on the COG data set.

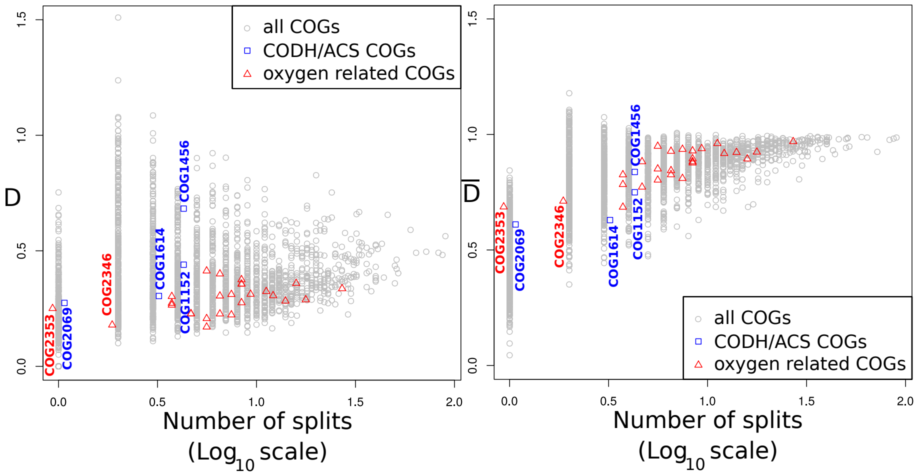

Figure 10: Values for D (left) and $\overline{D}$ (right) plotted against number of splits in log_{10} scale for the data set. COGs are gray, CODH/ACS COGs (blue) are those corresponding to the four subunits of CODH/ACS [1, 4] and oxygen related COGs are in red [5].

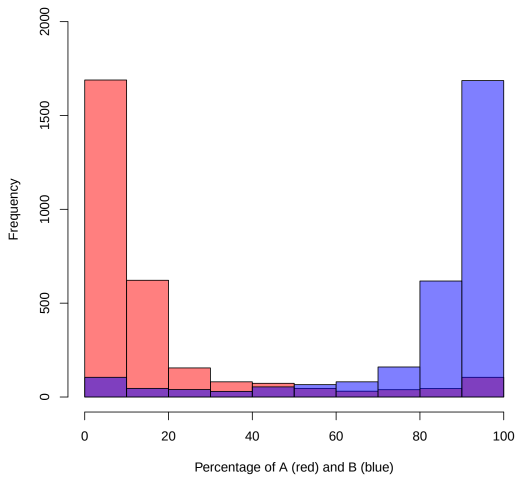

Figure 11: Plot showing frequencies of COGs with varying percentages of archaeal and bacterial seqeunces. The majority of COGs have a 10% - 90% distribution of archaeal (A, red) and bacterial (B, blue) proteins.

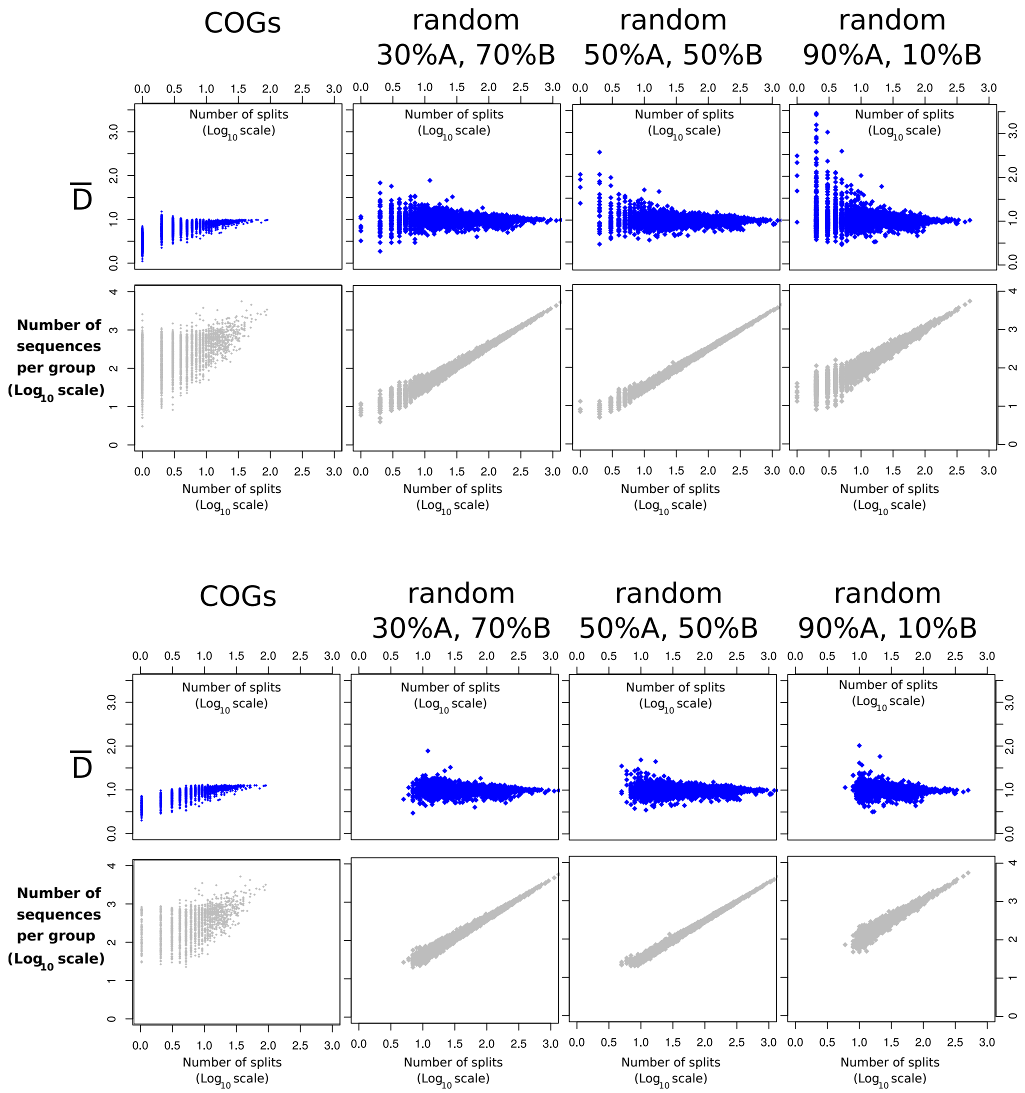

Figure 12: Plots comparing number of archaea:bacteria interdomain branches (splits) in log10 scale to $\overline{D}$ (blue) and the number of sequences per group in log10 scale (gray) for the set of COGs and randomized trees including all trees (top) and trees with at least 10 leaves of each label, a and b (bottom). Figure 11 shows distribution of COGs given various percentages of archaeal and bacterial species.

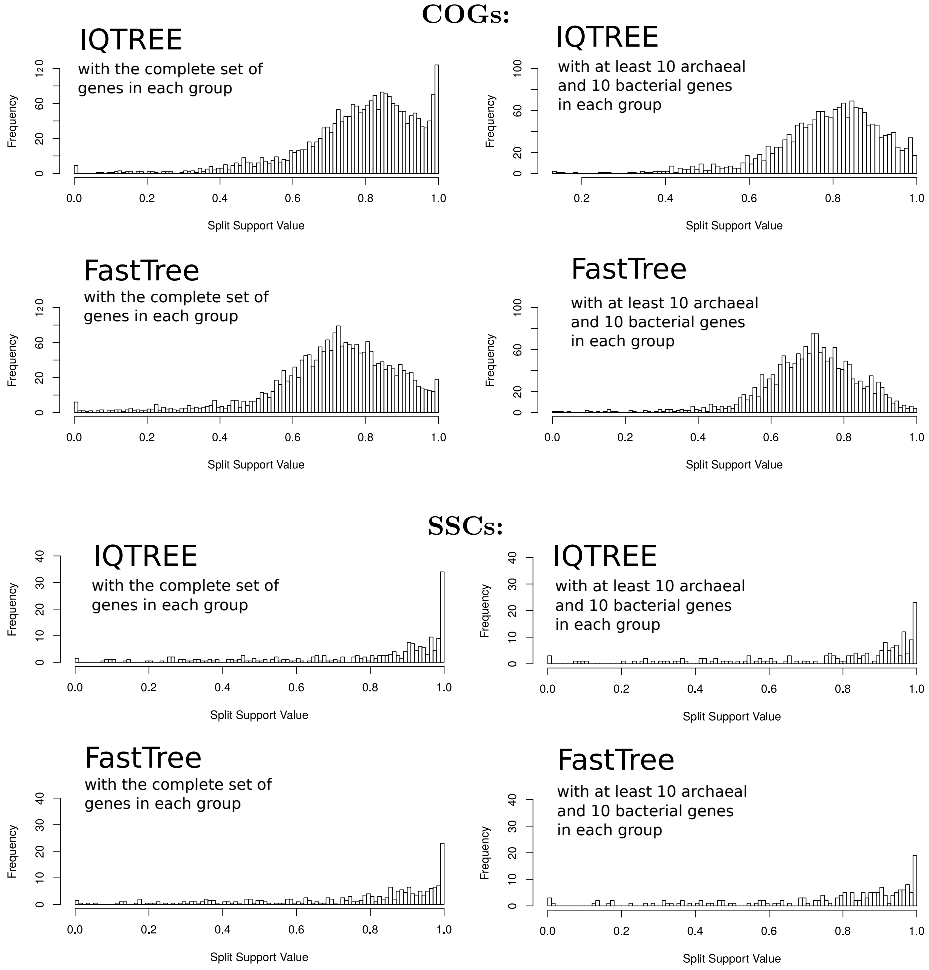

Figure 13: Histograms showing the distribution of support value at split nodes for the reconstructed phylogenetic trees for COGs (top) and SSCs (bottom). The trees were built with `IQTREE` [6] and `FastTree` [7]. Histograms on the left show support value distribution for the complete sets of COGs and SSCs. Histograms on the left only include groups containing at least 10 archaeal and 10 bacterial sequences.

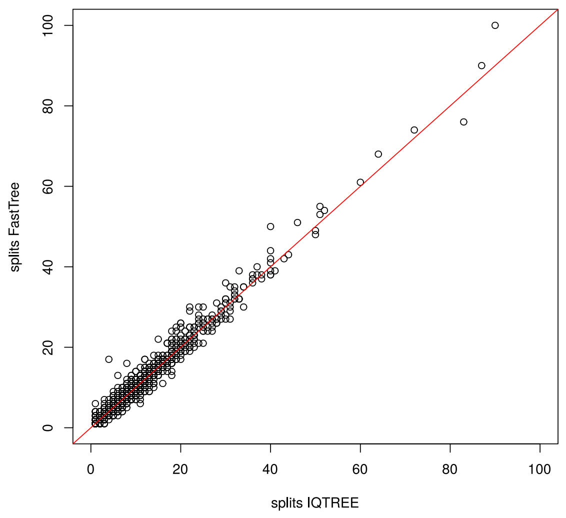

Figure 14: For each COG, the corresponding tree was reconstructed using `IQTREE` [6] and `FastTree` [7]. This plot depicts the number of interdomain splits between archaeal and bacterial sequences obtained from `IQTREE` and `FastTree` for each COGs. The red line shows the diagonal with a slope of 1. Different results for splits were obtained if the corresponding symbol in the plot is located apart from the red line.

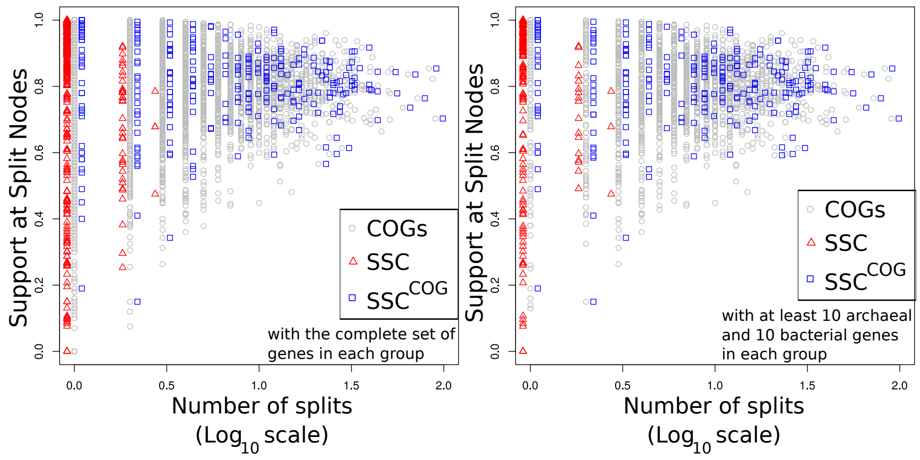

Figure 15: Plots comparing interdomain branch support values at split nodes and the number of splits in the tree, in log_{10}-scale for COGs, SSCs and SSC^{COG}. The plot on the left includes all groups of sequences whereas the plot on the right only include groups containing at least 10 archaeal and 10 bacterial sequences.

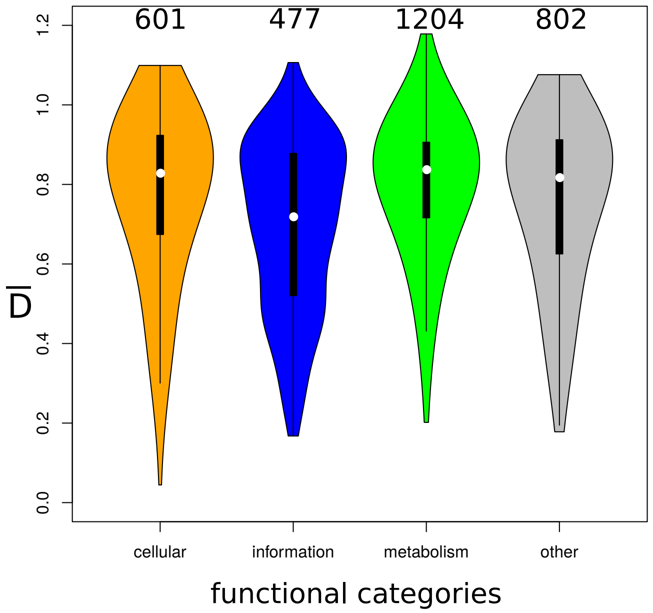

Figure 16: Violin plot showing distribution of $\overline{D}$ values for COGs and functional categories. The set COGs includes all groups with at least 1 archaeal and 1 bacterial sequence. Numbers on top of each violin indicate the total number of COGs in each group. In case a COG was associated with more than one category, it was counted in each.

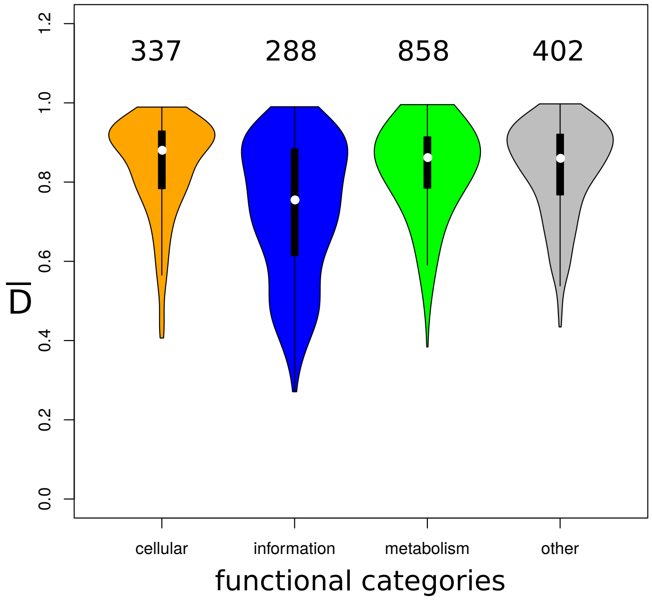

Figure 17: Violin plot showing distribution of $\overline{D}$ values for COGs and functional categories. The set COGs includes all groups with at least 10 archaeal and 10 bacterial proteins. Numbers on top of each violin indicate the total number of COGs in each group. In case a COG was associated with more than one category, it was counted in each.

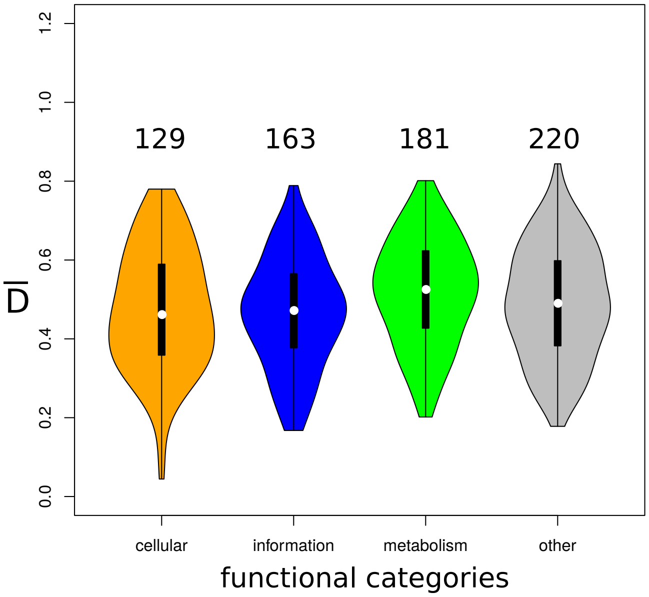

Figure 18: Violin plot showing distribution of $\overline{D}$ values for COGs and functional categories. The set COGs includes all groups where the corresponding tree shows a single split separating archaeal and bacterial proteins. Numbers on top of each violin indicate the total number of COGs in each group. In case a COG was associated with more than one category, it was counted in each.

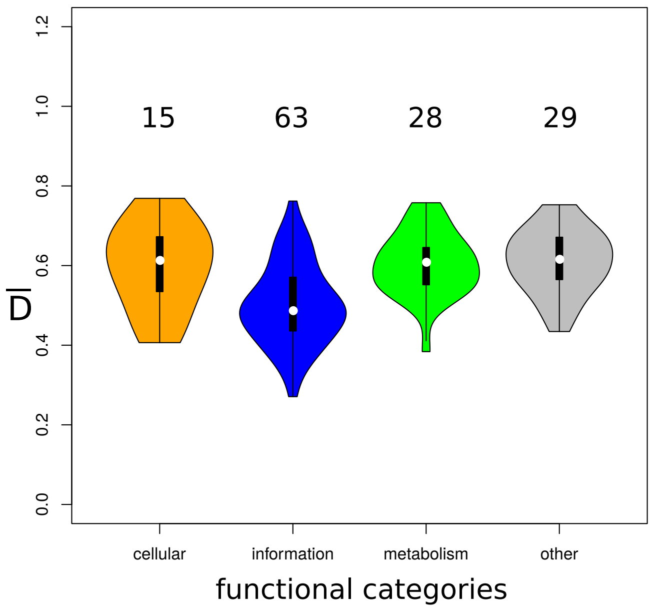

Figure 19: Violin plot showing distribution of $\overline{D}$ values for COGs and functional categories. The set COGs includes all groups where the corresponding tree shows a single split separating archaeal and bacterial proteins and additionally, all groups include at least 10 archaeal and 10 bacterial proteins. Numbers on top of each violin indicate the total number of COGs in each group. In case a COG was associated with more than one category, it was counted in each.

COG	Arc	Bac	Cat.	$\overline{D}$	Annotation
COG0008*	76	703	J	0.59	Glutamyl- or glutaminyl-tRNA synthetase
COG0012*	85	617	J	0.46	Ribosome-binding ATPase YchF, GTP1/OBG family
COG0051*	84	621	J	0.42	Ribosomal protein S10
COG0080*	83	632	J	0.50	Ribosomal protein L11
COG0081*	83	626	J	0.47	Ribosomal protein L1
COG0090*	83	624	J	0.44	Ribosomal protein L2
COG0093*	83	625	J	0.39	Ribosomal protein L14
COG0094*	83	625	J	0.39	Ribosomal protein L5
COG0096*	83	625	J	0.52	Ribosomal protein S8
COG0098*	83	626	J	0.43	Ribosomal protein S5
COG0100*	83	620	J	0.44	Ribosomal protein S11
COG0103*	83	626	J	0.49	Ribosomal protein S9
COG0130	19	603	J	0.65	tRNA U55 pseudouridine synthase TruB
COG0315	74	437	H	0.72	Molybdenum cofactor biosynthesis enzyme
COG0373	69	468	H	0.76	Glutamyl-tRNA reductase
COG0391	50	341	HG	0.53	Archaeal 2-phospho-L-lactate transferase
COG0466	9	534	O	0.58	ATP-dependent Lon protease, bacterial type
COG0480*	81	761	J	0.49	Translation elongation factor EF-G, a GTPase
COG0533*	42	617	J	0.59	tRNA A37 threonylcarbamoyltransferase TsaD
COG0541*	82	605	U	0.64	Signal recognition particle GTPase
COG0552*	79	586	U	0.66	Signal recognition particle GTPase
COG0643	1	38	NT	0.71	Chemotaxis protein histidine kinase CheA
COG0709	14	239	E	0.56	Selenophosphate synthase
COG0750	83	602	OK	0.66	Membrane-associated protease RseP
COG0805	47	523	U	0.74	Sec-independent protein secretion pathway component TatC
COG1027	7	215	E	0.69	Aspartate ammonia-lyase
COG1035	20	69	C	0.55	Coenzyme F420-reducing hydrogenase, beta subunit
COG1161	34	215	J	0.55	Ribosome biogenesis GTPase A
COG1162	7	500	J	0.58	Putative ribosome biogenesis GTPase RsgA
COG1163	83	10	J	0.67	Ribosome-interacting GTPase 1
COG1415	37	31	S	0.75	Uncharacterized protein
COG1777	47	2	K	0.67	Predicted transcriptional regulator
COG1836	61	71	S	0.71	Uncharacterized membrane protein
COG1851	4	4	S	0.54	Uncharacterized protein, UPF0128 family
COG1859	36	81	J	0.65	RNA:NAD 2'-phosphotransf., TPT1/KptA fam.
COG1883	6	146	C	0.68	Na+-transp. methylmalonyl-CoA/oxaloacetate decarboxylase
COG1915	36	59	S	0.67	Uncharacterized conserved protein
COG2032	2	187	P	0.76	Cu/Zn superoxide dismutase
COG2037	29	30	C	0.53	formyltransferase
COG2069	24	34	C	0.61	CO dehydrogenase/acetyl-CoA synthase delta subunit
COG2262	60	556	J	0.64	50S ribosomal subunit-associated GTPase HflX
COG2382	5	235	P	0.62	Enterochelin esterase or related enzyme
COG2920	5	139	P	0.59	Sulfur relay (sulfurtransferase) protein
COG3252	45	34	H	0.65	Methenyltetrahydromethanopterin cyclohydrolase
COG3376	7	72	P	0.66	High-affinity nickel permease
COG3482	36	59	S	0.59	Uncharacterized protein
COG3508	3	173	Q	0.62	Homogentisate 1,2-dioxygenase
COG3885	9	53	Q	0.56	Aromatic ring-opening dioxygenase, LigB subunit
COG4732	5	36	S	0.64	Predicted membrane protein
COG4754	22	67	S	0.58	Uncharacterized protein
COG4866	9	64	S	0.66	Uncharacterized protein
COG4962	13	377	UW	0.57	Pilus assembly protein, ATPase of CpaF family

Table 1: Data set of SSC^{COG} showing a single split. (*) indicates COGs that are in the set of single 3-domain split groups of Harris et al.

COG	s	Arc	Bac	Cat.	$\overline{D}$	Annotation
COG0109	4	30	402	HI	0.83	Polyprenyltransf.(heme O synthase)
COG0316	6	32	556	O	0.80	Fe-S cluster assembly iron-bind. prot. IscA
COG0400	7	18	385	R	0.83	Predicted esterase
COG0412	7	14	521	Q	0.93	Dienelactone hydrolase
COG0431	9	25	585	C	0.88	NAD(P)H-dependent FMN reductase
COG0569	19	76	661	P	0.92	Trk K+ transp. sys., NAD-bind. comp.
COG0604	13	39	1317	CR	0.92	NADPH:quinone or rel. Zn-dep. oxido reduct.
COG0654	6	9	1189	HC	0.95	2-polyprenyl-6-methoxyphenol hydroxylase
COG0665	15	47	984	E	0.92	Glycine/D-amino acid oxidase (deaminating)
COG0843	8	32	495	C	0.81	Heme/copper-type cytochrome/quinol oxidase, subunit 1
COG1012	29	76	1937	C	0.97	Acyl-CoA reduct. or other NAD-dep. dehyd.
COG1018	9	31	709	C	0.88	Ferredoxin-NADP reductase
COG1064	17	42	468	G	0.89	D-arabinose 1-dehydrogenase
COG1290	5	31	350	C	0.77	Cytochrome b subunit of the bc complex
COG1622	7	34	375	C	0.84	Heme/copper-type cytochrome/quinol oxidase, subunit 2
COG1764	4	12	420	V	0.78	Organic hydroperoxide reductase OsmC/OhrA
COG1845	5	24	496	C	0.88	Heme/copper-type cytochrome/quinol oxidase, subunit 3
COG2010	4	10	838	C	0.69	Cytochrome c, mono- and diheme variants
COG2128	6	18	601	P	0.85	Alkylhydroperoxidase family enzyme
COG2132	9	21	423	DPM	0.93	Multicopper oxidase with three cupredoxin domains
COG2133	10	28	581	G	0.94	Glucose/arabinose dehydrogenase, beta-propeller fold
COG2154	9	30	356	H	0.89	Pterin-4a-carbinolamine dehydratase
COG2259	8	20	530	S	0.94	Uncharact. membrane prot. YphA, DoxX/SURF4 fam.
COG2346	2	14	350	P	0.71	Truncated hemoglobin YjbI
COG2353	1	2	548	R	0.69	Polyisoprenoid-binding periplasmic protein YceI
COG4221	12	30	1124	C	0.96	NADP-dep. 3-hydroxy acid dehydrogenase YdfG
COG1152	4	23	18	C	0.75	CO dehydrogenase/acetyl-CoA synthase alpha subunit
COG1456	4	27	42	C	0.84	CO dehydrogenase/acetyl-CoA synthase gamma subunit
COG1614	3	24	36	C	0.63	CO dehydrogenase/acetyl-CoA synthase beta subunit
COG2069	1	24	34	C	0.61	CO dehydrogenase/acetyl-CoA synthase delta subunit
COG0085	1	87	619	K	0.42	DNA-directed RNA polymerase, beta/140 kD subunit
COG0086	1	82	537	K	0.39	DNA-directed RNA polymerase, beta'/160 kD subunit
COG0231	1	82	636	J	0.49	Transl. elong. f. P(EF-P)/transl. init. f. 5A(eIF-5A)
COG1110	2	35	20	L	0.84	Reverse gyrase

Table 2: Data for COGs appearing in the following data sets: 26 oxygen related COGs [5], 4 CODH/ACS COGs, 4 COGs mentioned by Catchpole & Forterre [2]. s:number of splits, cat:functional category.
